# Increased production of aureolysin and staphopain A is a primary determinant of the reduced virulence of *Staphylococcus aureus sarA* mutants in osteomyelitis

**DOI:** 10.1128/mbio.03383-23

**Published:** 2024-02-28

**Authors:** Mara J. Campbell, Karen E. Beenken, Aura M. Ramirez, Mark S. Smeltzer

**Affiliations:** 1 Department of Microbiology and Immunology, University of Arkansas for Medical Sciences, Little Rock, Arkansas, USA; MedImmune, Gaithersburg, Maryland, USA

**Keywords:** *Staphylococcus aureus*, osteomyelitis, *sarA*, proteases, biofilm, cytotoxicity

## Abstract

We previously demonstrated that mutation of *sarA* in *Staphylococcus aureus* limits biofilm formation, cytotoxicity for osteoblasts and osteoclasts, and virulence in osteomyelitis, and that all of these phenotypes can be attributed to the increased production of extracellular proteases. Here we extend these studies to assess the individual importance of these proteases alone and in combination with each other using the methicillin-resistant USA300 strain LAC, the methicillin-susceptible USA200 strain UAMS-1, and isogenic *sarA* mutants that were also unable to produce aureolysin (Aur), staphopain A (ScpA), staphylococcal serine protease A (subsp.), staphopain B (SspB), and the staphylococcal serine protease-like proteins A-F (SplA-F). Biofilm formation was restored in LAC and UAMS-1 *sarA* mutants by subsequent mutation of *aur* and *scpA*, while mutation of *aur* had the greatest impact on cytotoxicity to mammalian cells, particularly with conditioned medium (CM) from the more cytotoxic strain LAC. However, SDS-PAGE and western blot analysis of CM confirmed that mutation of *sspAB* was also required to mimic the phenotype of *sarA* mutants unable to produce any extracellular proteases. Nevertheless, in a murine model of post-traumatic osteomyelitis, mutation of *aur* and *scpA* had the greatest impact on restoring the virulence of LAC and UAMS-1 *sarA* mutants, with concurrent mutation of *sspAB* and the *spl* operon having relatively little effect. These results demonstrate that the increased production of Aur and ScpA in combination with each other is a primary determinant of the reduced virulence of *S. aureus sarA* mutants in diverse clinical isolates including both methicillin-resistant and methicillin-susceptible strains.

**IMPORTANCE:**

Previous work established that SarA plays a primary role in limiting the production of extracellular proteases to prevent them from limiting the abundance of *S. aureus* virulence factors. Eliminating the production of all 10 extracellular proteases in the methicillin-resistant strain LAC has also been shown to enhance virulence in a murine sepsis model, and this has been attributed to the specific proteases Aur and ScpA. The importance of this work lies in our demonstration that the increased production of these same proteases largely accounts for the decreased virulence of *sarA* mutants in a murine model of post-traumatic osteomyelitis not only in LAC but also in the methicillin-susceptible human osteomyelitis isolate UAMS-1. This confirms that *sarA*-mediated repression of Aur and ScpA production plays a critical role in the posttranslational regulation of *S. aureus* virulence factors in diverse clinical isolates and diverse forms of *S. aureus* infection.

## INTRODUCTION

**Fig 1:**
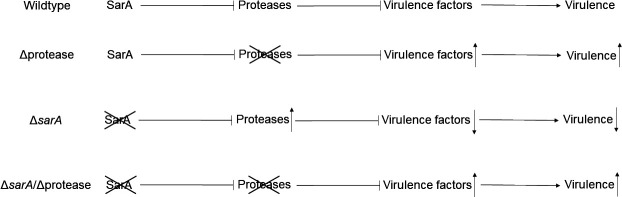
Schematic illustrating the relationship between SarA, extracellular proteases, and virulence. In wildtype *S. aureus*, SarA represses the production of extracellular proteases, some or all of which posttranslationally limit the balancing and abundance of virulence factors, thus influencing virulence. Mutation of the genes encoding these proteases results in the increased abundance of virulence factors and hypervirulence. Mutation of *sarA* eliminates this SarA-mediated protease repression of proteases production, resulting in the degradation of virulence factors and decreased virulence. Concomitant mutation of both *sarA* and the genes encoding extracellular proteases results in increased abundance of virulence factors, resulting in a virulence phenotype that more closely mimics the wildtype phenotype despite mutation of *sarA*.

Previous studies from our laboratory established that a critical function of the *sarA*-regulatory pathway is to limit the production of *Staphylococcus aureus* extracellular proteases such that they serve their beneficial purposes on behalf of the bacterium without limiting the abundance of *S. aureus* virulence factors (1–8). These studies were based on comparisons between staphylococcal accessory regulator A (*sarA*) mutants and isogenic derivatives unable to produce any of the 10 primary *S. aureus* extracellular proteases, specifically aureolysin (Aur), staphopain A (ScpA), staphylococcal serine protease A (V8 protease, subsp.) staphopain B (SspB), and the serine protease-like proteins (SplA-F) (5–7, 9–11). Collectively, these studies demonstrate that mutation of *sarA*, which encodes the highly conserved transcriptional regulator SarA (12), increases expression of the genes encoding these proteases, limits the abundance of over 1,000 *S*. *aureus* proteins present in conditioned medium (CM) from overnight cultures and, more importantly, limits virulence in murine models of sepsis, implant-associated infection, and osteomyelitis (2–8). This work also confirmed that eliminating the production of these 10 extracellular proteases restores the abundance of most of these *S. aureus* proteins and increases the virulence of *sarA* mutants to levels comparable to the parent strain in these same animal models (Fig. 1), indicating that the increased production of some or all of these proteases must be responsible for the reduced virulence of *sarA* mutants. Studies from other laboratories have demonstrated that eliminating the production of proteases in the methicillin-resistant *S. aureus* (MRSA) USA300 clinical isolate LAC (Los Angeles County clone) results in increased accumulation of virulence factors and hypervirulence (Fig. 1) in a murine sepsis model, specifically due to the inability to produce Aur and ScpA (9–11).

Because mutation of *sarA* results in an increase in the abundance of all 10 extracellular proteases (2, 6, 7), it remains unknown whether specific proteases also play a primary role in the attenuation of *sarA* mutant osteomyelitis or any of the protease-dependent *sarA* mutant phenotypes identified (3–6, 13–15). Given the large number of full-length proteins that are present in reduced amounts in CM from *sarA* mutants due to protease-mediated degradation (2, 7), and based on the presumption that this includes *S. aureus* proteins that contribute to these phenotypes, determining which of the dysregulated proteases is responsible for attenuation could prove invaluable in identifying specific virulence factors of importance for various forms of *S. aureus* infection including osteomyelitis. For instance, mutation of the *saePQRS* (*sae*) operon has been shown to result in the increased production of Aur, which limits the abundance of alpha-type phenol-soluble modulins (PSMs) to a degree that can be correlated with reduced cytotoxicity and reduced cortical bone destruction (16). We extended these studies to compare 6 isogenic derivatives of LAC that differed from each other based on the functional status of *sae* and *sarA* relative to each other and identified 114 *S*. *aureus* proteins that differed in abundance in a fashion that could be correlated with relative virulence in an osteomyelitis model (7). Finally, the sepsis study demonstrating that Aur and ScpA are responsible for the hypervirulence of LAC protease mutants identified several potentially important virulence factors including an uncharacterized secreted protein (SAUSA300_0964) that had not been previously associated with virulence in any context (10).

Thus, in this report, we extend upon previous work to determine whether the increased production of specific proteases is responsible for the attenuation of *sarA* mutants in the clinical context of osteomyelitis. To this end, we generated derivatives of *S. aureus sarA* mutants that were unable to produce individual proteases alone and in combination and examined these mutants for differences in important *in vitro* phenotypes implicated in the pathogenesis of osteomyelitis, and relative virulence in a murine osteomyelitis model. We did these experiments in both LAC and the USA200, methicillin-susceptible osteomyelitis isolate UAMS-1. LAC and UAMS-1 were chosen based on their clinical provenance and their established dissimilarity in both methicillin susceptibility and other phenotypes. For instance, unlike LAC, UAMS-1 does not encode the regulatory loci *sarT* or *sarU* (17–20). LAC also produces both alpha toxin and Panton-Valentine Leukocidin (PVL, LukSF), while UAMS-1 does not, and UAMS-1 produces the collagen-binding adhesin Cna, while LAC does not (17–19, 21, 22). Lastly, while the *spl* operon is fully intact (*splA-F*) in LAC, it is truncated (*splC-F*) in UAMS-1 (17–20).

## RESULTS

### Aur and ScpA are the primary proteases that define *in vitro* phenotypes of *sarA* mutants

**Fig 2:**
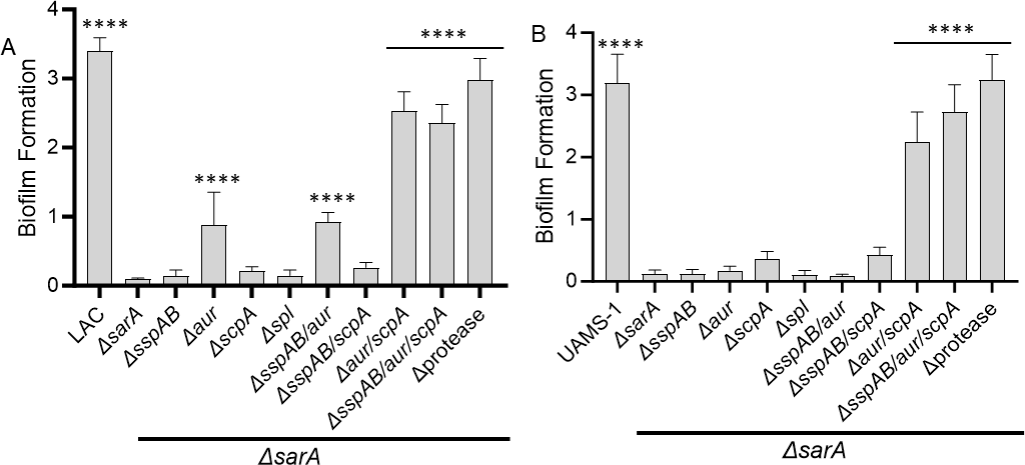
Increased production of Aur and ScpA plays a primary role in defining the biofilm formation of *S. aureus sarA* mutants. Biofilm formation was assessed with LAC (left), UAMS-1 (right), and isogenic *sarA* mutants unable to produce specific proteases alone and in combination. Biofilm formation was assessed by staining with crystal violet and measuring the absorbance at 595 nm. Statistical analysis was done using a one-way ANOVA with Dunnett’s correction. Asterisks indicate a statistically significant difference against the corresponding *sarA* mutant (****, *P* < 0.0001).

As expected based on our previous work, mutation of *sarA* essentially abolished biofilm formation in both LAC and UAMS-1, and subsequently eliminating the production of all extracellular proteases (protease/*sarA*) restored biofilm formation (Fig. 2). Mutation of *aur* significantly enhanced biofilm formation in a LAC *sarA* mutant (Fig. 2A) but had little impact in a UAMS-1 *sarA* mutant (Fig. 2B). Mutation of *scpA* did not have a significant impact in either strain. However, in both strains, mutation of *aur* and *scpA* was sufficient to restore biofilm formation in the *sarA* mutant to nearly the same level as the parental strain (*P* < 0.001) (Fig. 2). Eliminating the production of all proteases further enhanced biofilm formation to a significant extent (LAC *aur/scpA/sarA* vs protease/*sarA, P* = 0.0006; UAMS-1 *aur/scpA/sarA* vs protease/*sarA P* = <0.0001), although the increase was modest in comparison to that observed with the isogenic *aur*/*scpA*/*sarA* mutants (Fig. 2). In UAMS-1, a significant difference was also observed between the *aur*/*scpA*/*sarA* and *sspAB*/*aur*/*scpA*/*sarA* mutants (*P* = 0.0109) (Fig. 2B).

**Fig 3:**
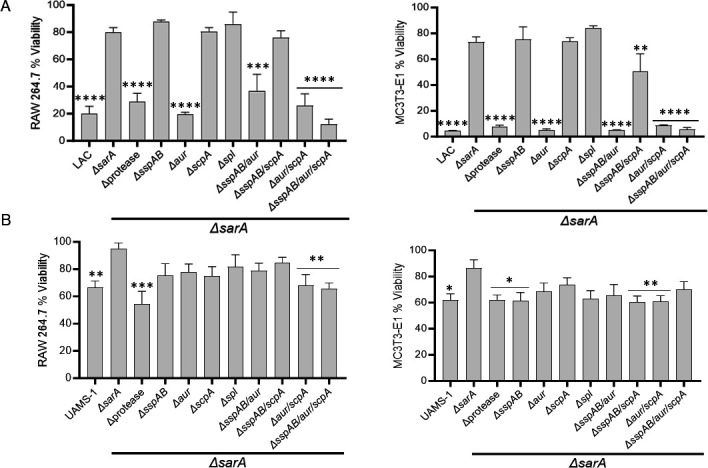
Increased production of Aur and ScpA plays a primary role in defining the cytotoxicity of *S. aureus sarA* mutants. Cytotoxicity of conditioned medium (CM) from overnight cultures was assessed against RAW 264.7 cells (left) and MC3T3-E1 cells (right) with LAC (**A**), UAMS-1 (**B**), and their isogenic *sarA* mutants unable to produce specific proteases alone and in combination. Results are reported as the percent (%) viability of the mammalian cells after 24 hours of exposure to CM as determined by fluorescent LIVE/DEAD staining, with the average fluorescence of a sterile media exposed control set as 100% viability. Statistical analysis was done using a one-way ANOVA with Dunnett’s correction. Asterisks indicate a statistically significant change against the corresponding *sarA* mutant (*, *P* < 0.05; **, *P* < 0.01; ***, *P* < 0.001; ****, *P* < 0.0001).

The cytotoxicity of conditioned media (CM) from overnight cultures was assessed using RAW246.7 and MC3T3-E1 cells as surrogates for osteoclasts and osteoblasts, respectively. Cytotoxicity was greater with CM from LAC than from UAMS-1 (Fig. 3), which made changes associated with mutation of *sarA* and specific protease genes more evident in LAC than UAMS-1. In LAC, mutation of *sarA* limited cytotoxicity for both cell types to a significant degree, and this was reversed by mutation of *aur* alone (Fig. 3A), but in UAMS-1, mutation of both *aur* and *scpA* was necessary to restore cytotoxicity to a level comparable to protease/*sarA* mutant (Fig. 3B). Mutation of *sspAB* also had an effect in UAMS-1, though inconsistently. Cytotoxicity against RAW264.7 cells was not significantly impacted by mutation of *sspAB*, but it was observed in MC3T3-E1 cells for some *sspAB* mutants (UAMS-1 s*spAB/sarA* vs *sarA, P* = 0.0494; *sspAB/scpA/sarA* vs *sarA, P* = 0.0351) (Fig. 3B). However, these differences should be interpreted with caution given the limited cytotoxicity of UAMS-1 CM, and overall these results are still consistent with the hypothesis that mutation of *aur/scpA* has the most significant effect on cytotoxicity even in UAMS-1.

### SspAB contributes to the degradation of secreted proteins in *sarA* mutants

**Fig 4:**
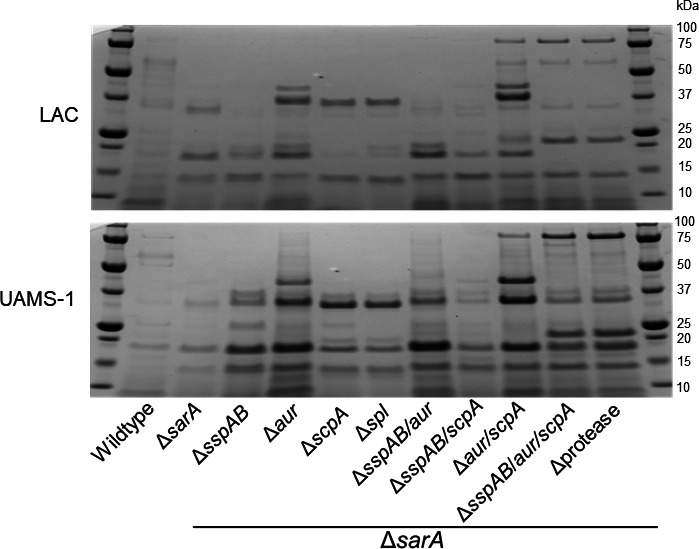
Mutation of *aur*, *scpA*, and *sspAB* is required to fully mimic the *in vitro* protein profiles of a protease/*sarA* mutant. SDS-page gels were run with CM of the designated wildtype strain, isogenic *sarA* mutants, and *sarA* mutants unable to produce all (Δprotease) or the designated extracellular proteases. Coomassie staining was performed for visualization of total exoprotein profiles. Molecular weight is indicated to the right of each panel.

The results discussed above implicate Aur and ScpA as the primary determinants of osteomyelitis-associated *in vitro* phenotypes in *S. aureus sarA* mutants. However, when we shifted the experimental focus to specific virulence factors implicated in these phenotypes, we found that it was necessary to also mutate the *sspAB* operon to fully mimic the phenotype of LAC and UAMS-1 protease/*sarA* mutants. Specifically, SDS-PAGE analysis confirmed the loss of multiple high molecular weight proteins in CM from both LAC and UAMS-1 *sarA* mutants, and the protease/*sarA* mutant had increased abundance of these proteins even by comparison to the parental strain. High molecular weight proteins are first restored in the *aur/scpA/sarA* mutant, but the overall protein profile of the *sarA* mutant unable to produce Aur, ScpA, and SspA/B was the most similar to that observed in protease/*sarA* mutant (Fig. 4).

**Fig 5:**
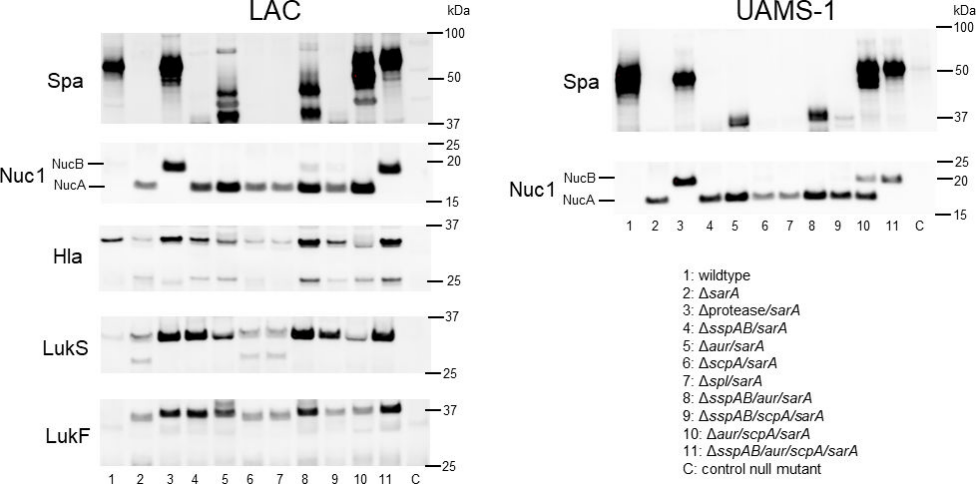
Mutation of *aur*, *scpA*, and *sspAB* is required to fully restore the abundance of specific virulence factors implicated in biofilm formation and cytotoxicity. Western blots of CM were performed for specific virulence factors with antibodies for staphylococcal protein A (Spa), extracellular nuclease (Nuc1), alpha-toxin (Hla), and both components of the Panton-Valentine leukocidin (LukS and LukF). NucA and NucB are the truncated and full-length forms of Nuc1, respectively. The control null mutants (**C**) are isogenic mutants unable to express the virulence factors of interest. Molecular weight is indicated to the right of each panel.

The impact of SspA/B was also evident in western blots focusing on the specific virulence factors implicated in biofilm formation and cytotoxicity. Specifically, in a LAC *sarA* mutant, protein A (Spa), nuclease (Nuc1), and its truncated (NucA) or full-length forms (NucB), alpha toxin, and both components, LukS and LukF, of the toxin PVL were all either absent in the case of Spa and alpha toxin or present in a truncated form in the case of Nuc1, LukS, and LukF. Restoration of the full-length form of these virulence factors required subsequent mutation of *aur*, *scpA*, and *sspAB* (Fig. 5). UAMS-1 does not produce alpha toxin or either PVL component, but the absence of full-length Spa and NucB in the UAMS-1 *sarA* mutant also required mutation of *aur*, *scpA*, and *sspAB* to reverse (Fig. 5).

### Contribution of specific extracellular proteases to the attenuation of a LAC *sarA* mutant in osteomyelitis

**Fig 6:**
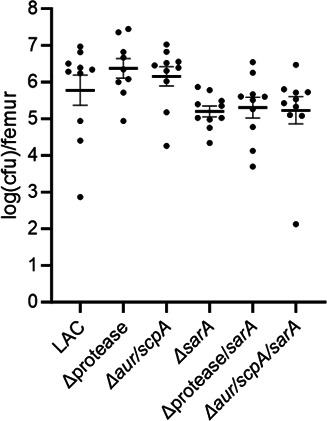
Impact of *sarA* and extracellular proteases on bacterial burdens in the femur in mice infected with LAC. C57BL/6 mice (*n* = 5 per group, two biological replicates) were infected with LAC, its *sarA* mutant (Δ*sarA*), or derivatives unable to produce either Aur and ScpA (Δ*aur/scpA* and Δ*aur/scpA/sarA*) or any extracellular protease (Δprotease and Δprotease/*sarA*). After 14 days, bones were harvested, imaged by μCT, and homogenized for serial dilutions and plate counts to determine bacterial burden, which is reported as the logarithmically transformed colony-forming units (cfu) counted for each femur. Statistical analysis was done by one-way ANOVA with Dunnett’s correction. Comparison against LAC yielded no significant differences.

Based on the impact of Aur and ScpA on multifactorial phenotypes associated with the pathogenesis of osteomyelitis and previous work demonstrating that eliminating the ability to produce Aur and ScpA was both necessary and sufficient for hypervirulence in LAC (10, 11), we first used our murine osteomyelitis model to assess the relative virulence of LAC and its protease, *aur/scpA, sarA,* protease/*sarA,* and *aur/scpA/sarA* mutants. At 14 days postinfection, the femur was harvested for micro-computed tomography (µCT) imaging and determination of bacterial burden. There were no statistically significant differences against the bacterial burden of mice infected with LAC, but there was a statistically significant difference from the *sarA* mutant group against the protease mutant group (*P* = 0.0396), supporting an upward trend in the protease deficient derivatives and a downward trend in the *sarA* mutant (Fig. 6).

**Fig 7:**
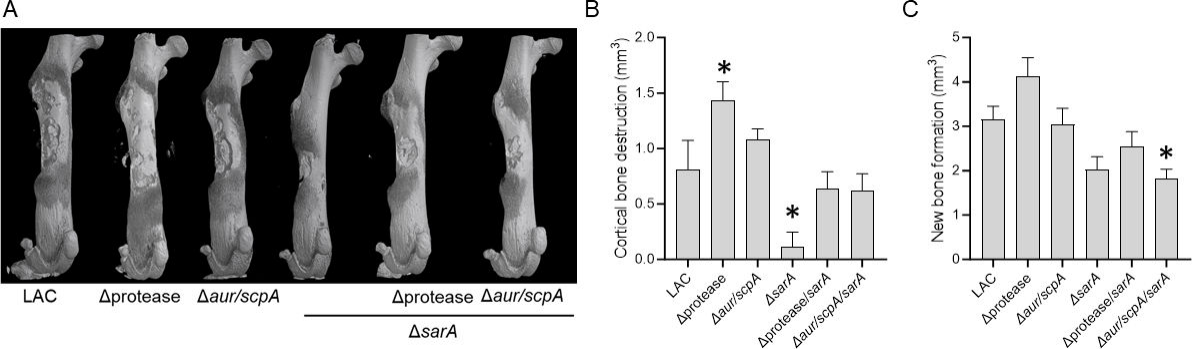
Mutation of *aur* and *scpA* enhances virulence in LAC and its *sarA* mutant. Bones from C57BL/6 mice infected with LAC, Δprotease, Δ*aur/scpA*, Δ*sarA*, Δprotease/*sarA*, and Δ*aur/scpA/sarA* were harvested for μCT imaging (**A**) and quantitative analysis of cortical bone destruction (**B**) and new bone formation (**C**). Randomly selected bones were used to generate representative μCT images (**A**). Statistical analysis was done by one-way ANOVA with Dunnett’s correction. Asterisks indicate statistical significance against the LAC-infected group (*, *P* ≤ 0.05).

Similar trends were visually apparent in randomized representative µCT images of mice from each experimental group, with cortical bone destruction and new bone formation appearing to be increased in mice infected with protease-deficient derivatives of LAC and decreased in mice infected with the *sarA* mutants (Fig. 7A). Visual inspection also suggested a comparable increase in cortical bone destruction in mice infected with the *aur*/*scpA*/*sarA* mutant and mice infected with the protease/*sarA* mutant, which was confirmed by quantitative µCT analysis. Specifically, relative to LAC, the *sarA* mutant had significantly less cortical bone destruction (*P* = 0.0158) while the protease mutant had significantly more cortical bone destruction (*P* = 0.0495) (Fig. 7B). Relative to the *sarA* mutant, cortical bone destruction trended upward to a comparable degree in the *aur/scpA/sarA* mutant (*P* = 0.0907) and protease/*sarA* mutant infected groups (*P* = 0.0752), and the differences between these two groups or in comparison to the parental strain were not significant (Fig. 7B). In contrast, new bone formation was more similar to bacterial burdens, with a statistically significant difference between mice infected with the LAC protease mutant relative to mice infected with the *sarA* mutant (*P* = 0.0002) but not between mice infected with the *sarA* mutant and any other group, although the *aur/scpA* mutant also trended upward (*P* = 0.0925) (Fig. 7C). In comparison to the parental strain, the *sarA* mutant group trended downward (*P* = 0.0673), and the *aur/scpA/sarA* mutant group was significantly different (*P* = 0.0250).

**Fig 8:**
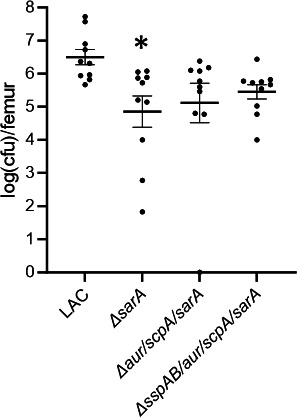
Impact of *sarA* and extracellular proteases on bacterial burdens in the femur in mice infected with LAC. C57BL/6 mice (*n* = 5 per group, two biological replicates) were infected with LAC and its isogenic Δ*sarA*, Δ*aur/scpA/sarA* and *ΔsspAB*/*aur/scpA/sarA* mutants. After 14 days, bones were harvested, imaging by μCT, and homogenized for serial dilutions and plate counts to determine bacterial burden, which is reported as the logarithmically transformed colony-forming units (cfu) obtained from each femur. Statistical analysis was performed by one-way ANOVA with Dunnett’s correction. Asterisks indicate statistical significance against the LAC-infected group (*, *P* < 0.05).

The results of this first *in vivo* experiment suggested that the increased production of Aur and ScpA is important in defining the attenuation of a LAC *sarA* mutant, and that the inability to produce these proteases enhances the virulence of LAC itself in our osteomyelitis model. However, the statistical analysis was limited by variability within groups, and, when viewed collectively and in the context of our *in vitro* results, these results did not rule out the possibility that other proteases are also involved, particularly SspA or SspB. To address this issue, we directly compared mice infected with LAC and its *sarA*, *aur*/*scpA*/*sarA*, and *sspAB/aur*/*scpA/sarA* mutants. The only significant difference in bacterial burdens by comparison to LAC was the *sarA* mutant (*P* = 0.0206). The observation that the differences between the *aur/scpA*/*sarA* and *sspAB*/*aur*/*scpA*/*sarA* mutants by comparison to LAC were not significant (*P* = 0.1909 and 0.0589, respectively) suggests an upward trend associated with eliminating specific proteases, including Aur and ScpA, in the *sarA* mutant (Fig. 8).

**Fig 9:**
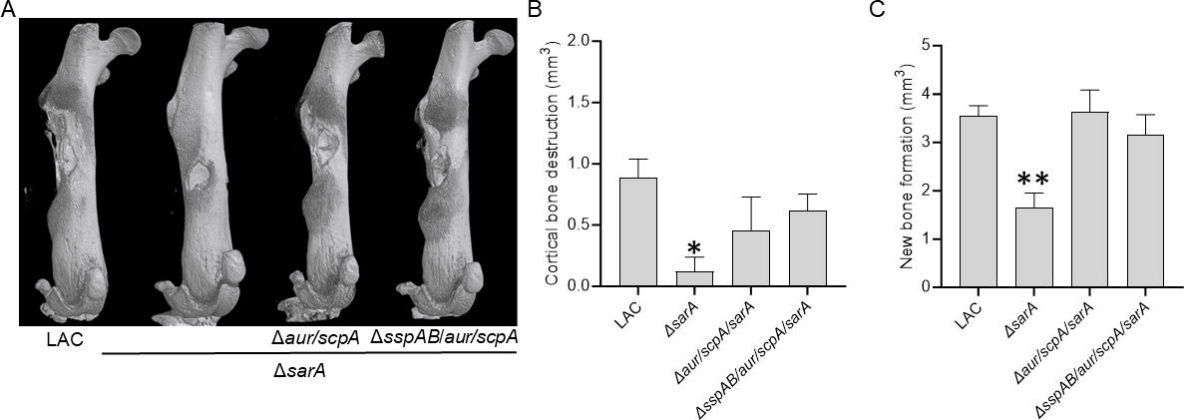
Mutation of *sspAB/aur/scpA*/*sarA* in LAC does not enhance virulence beyond that of an *aur/scpA/sarA* mutant. Bones from C57BL/6 mice infected with LAC and its isogenic Δ*sarA*, Δ*aur/scpA/sarA* and Δ*aur/scpA/sspAB/sarA* mutants were harvested at 14-day postinfection for μCT imaging (**A**) and analysis of cortical bone destruction (**B**) and new bone formation (**C**). Randomly selected bones were used to generate representative μCT images (**A**). Statistical analysis was done by one-way ANOVA with Dunnett’s correction. Asterisks indicate statistical significance against the LAC-infected group (*, *P* < 0.05, **, *P* < 0.01).

These same trends were evident in the visual examination of µCT images (Fig. 9A) and, as with bacterial burdens in the femur, the only significant difference in cortical bone destruction (Fig. 9B) and new bone formation (Fig. 9C) was between mice infected with LAC and mice infected with its *sarA* mutant (*P* = 0.0163 and 0.0026, respectively). However, there was an upward trend for groups infected with the *aur/scpA/sarA* and *sspAB/aur/scpA/sarA* mutants when compared to the *sarA* mutant group, particularly in the case of new bone formation where these differences were statistically significant (*P* = 0.0012 and 0.0141, respectively) (Fig. 9C). Thus, while we cannot completely rule out the possibility that increased production of SspA and/or SspB contribute to the attenuation of *sarA* mutants, the results of this experiment support the hypothesis that the contribution of these proteases is minor in comparison to Aur and ScpA.

### Contribution of specific extracellular proteases to the attenuation of a UAMS-1 *sarA* mutant

**Fig 10:**
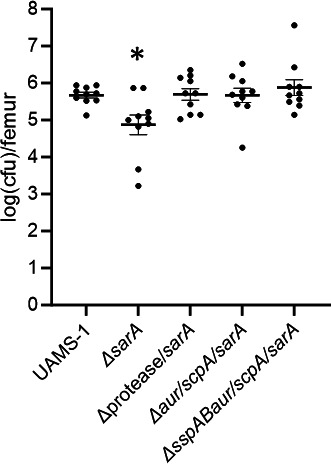
Impact of *sarA* and extracellular proteases on bacterial burdens in the femur in mice infected with UAMS-1. C57BL/6 mice (*n* = 5 per group, two biological replicates) were infected with UAMS-1, its *sarA* mutant (Δ*sarA*), a *sarA* mutant also unable to produce any extracellular proteases (Δprotease/sarA), or the Δ*aur/scpA/sarA* and Δ*sspAB/aur/scpA/sarA* mutants. After 14 days, bones were harvested, imaged by μCT, and homogenized for serial dilutions and plate counts to determine bacterial burden. Results are reported as the logarithmically transformed colony-forming units (cfu) obtained from each femur. Statistical analysis was performed by one-way ANOVA with Dunnett’s correction. Asterisks indicate statistical significance against the UAMS-1 infected group (*, *P* < 0.05).

In order to include genotypically and phenotypically diverse clinical isolates of *S. aureus*, we took a targeted approach to determine whether Aur and ScpA are also primary determinants of the reduced virulence of a UAMS-1 *sarA* mutant. As in LAC, a statistically significant difference was observed in the bacterial burdens between the UAMS-1 infected group and the *sarA* mutant group (*P* = 0.0177), but not any other group. Moreover, relative to the *sarA* mutant, the protease/*sarA*, *aur/scpA/sarA*, and *sspAB/aur/scpA/sarA* mutant infected groups were all significantly increased (*P* = 0.0144, 0.0189, and 0.0022, respectively) (Fig. 10).

**Fig 11:**
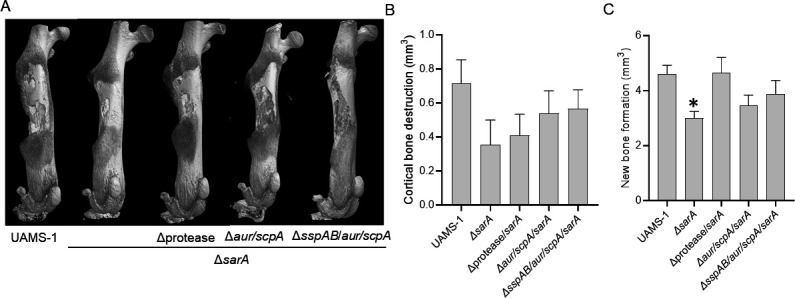
Mutation of *sspAB* does not enhance the virulence of a UAMS-1 *sarA* mutant beyond that of a UAMS-1 *aur/scpA/sarA* mutant. C57BL/6 mice (*n* = 5 per group, two biological replicates) were infected with UAMS-1, its *sarA* mutant (Δ*sarA*), *sarA* mutants unable to produce any extracellular protease (Δprotease/*sarA*), and *sarA* mutants unable to produce either Aur and ScpA (Δ*aur/scpA/sarA)* or Aur, ScpA, and SspAB (Δ*sspAB*/*aur/scpA/sarA*). After 14 days, bones were harvested for μCT imaging (**A**) and quantitative analysis of cortical bone destruction (**B**) and new bone formation (**C**). Randomly selected bones were used to generate the representative μCT images (**A**). Statistical analysis was performed by one-way ANOVA with Dunnett’s correction. Asterisks indicate statistical significance against the UAMS-1 infected group (*, *P* < 0.05).

The µCT analysis results for cortical bone destruction and new bone formation were less clear (Fig. 11). Specifically, there were no significant differences in cortical bone destruction in comparison to groups infected with the parent strain or the *sarA* mutant (Fig. 11B), and in new bone formation, the only significant difference relative to the parent strain infected group was between the UAMS-1 and the *sarA* mutant infected groups (*P* = 0.0320). The *sarA* mutant group was also significantly different compared to the protease/*sarA* mutant (*P* = 0.0267). However, in both measures of virulence, the protease/*sarA* mutant was also not significantly different from either of the other protease-deficient derivatives. Therefore, this result is consistent with the hypothesis that the increased production of proteases plays a key role in defining the attenuation of *sarA* mutants, and that the primary proteases responsible for this attenuation are Aur and ScpA.

## DISCUSSION

Osteomyelitis and other forms of orthopedic infection are remarkably recalcitrant to conventional antibiotic therapy irrespective of the resistance status of the offending strain (23, 24). It is our hope that investigating the role of extracellular proteases in virulence regulation in the context of osteomyelitis will allow the development of alternative strategies to overcome this therapeutic recalcitrance. While a number of *Staphylococcus aureus* regulatory loci have been shown to modulate protease production, our data provides strong support for the hypothesis that *sarA* plays the predominant role in this regard. For instance, using an unbiased protein capture approach with protease promoters as bait, we identified six *S. aureus* regulatory proteins that bind most if not all of these promoters, but SarA was clearly the most abundant (6). Mutation of *sarA* resulted in a greater increase in protease production than mutation of the loci encoding any of these other regulatory proteins, which suggests that *sarA* is the major regulator of extracellular proteases and that *sarA*-mediated repression of protease production occurs via a direct interaction between SarA and its DNA targets (6). Moreover, we recently extended these studies to demonstrate that mutation of *sarA* limits the virulence of LAC and UAMS-1 in our osteomyelitis model to a greater extent than mutation of any of the loci encoding these other regulatory proteins (3).

Therefore, one mechanism-independent option for the development of alternative therapeutics is to identify inhibitors of *sarA* expression and/or function. Since mutation of *sarA* has long been known to inhibit biofilm formation, cytotoxicity and virulence in osteomyelitis (4, 6, 8, 15, 25, 26), several reports have already described the identification of such inhibitors with a primary focus on limiting biofilm formation (27–32). However, we recently examined a number of these putative inhibitors using the same biofilm assay used in this report, and we failed to identify any that effectively inhibited biofilm formation to the same degree as mutation of *sarA* in LAC and UAMS-1 (33).

An alternative approach is to use the inverse correlation between increased protease production and decreased virulence in our osteomyelitis model to identify specific *S. aureus* proteins that warrant further investigation. For instance, mutation of *saePQRS* (*sae*) or *sarA* results in increased protease production, decreased biofilm formation, and decreased virulence in our murine osteomyelitis model (6, 8, 14, 16, 26). Based on this effect, we generated 5 derivatives of LAC that differed based on the functional status of *sae* and *sarA* relative to each other and assessed the impact on virulence and overall protein profiles, which led to the identification of 114 proteins that were present in significantly greater amounts (log_2_ fold-change >2.0) in virulent versus attenuated strains as defined by our osteomyelitis model (7).

The question then became how to prioritize among these 114 proteins. We first attempted to prioritize by applying a more stringent standard for significance. For instance, of these 114 proteins, 14 were present in virulent strains at levels that were log_2_ fold-change >5.0 higher than the levels observed in the attenuated strains (7). Alternatively, another approach is to layer in additional mutants and strains that exhibit significant differences in virulence. As an example, one of the 14 proteins we identified is Sbi (7) was also identified in the studies investigating the hypervirulence of LAC protease mutants (10). Because our studies demonstrating the importance of extracellular proteases in defining *in vitro* and *in vivo* phenotypes of *S. aureus sarA* mutants were done with *sarA* mutants which were unable to produce any of the 10 primary extracellular proteases, a third approach, and the approach this report begins to address, is to determine whether specific proteases are particularly important and, if so, to focus on the targets of these proteases.

In experiments focusing on LAC, Aur and ScpA were found to be key determinants of the hypervirulence of protease mutants in a murine sepsis model (10, 11). Therefore, we aimed to determine whether Aur and ScpA or any of the other proteases were specifically responsible for the attenuation of *sarA* mutants in osteomyelitis. We also aimed to take the diversity among *S. aureus* clinical isolates into account by using mutants generated in both LAC and UAMS-1.

Despite their differences, mutation of *sarA* limits the virulence of both strains in multiple animal models, and this attenuation can be attributed to a significant extent to the increased production of extracellular proteases (3, 5–8, 15). This is not to discount the importance of *sarA* as a transcriptional regulator of other *S. aureus* genes; indeed, eliminating the ability of *sarA* mutants to produce extracellular proteases did not entirely restore virulence in our osteomyelitis model, as new bone formation and cortical bone destruction values for mice infected with the protease/*sarA* mutants were generally lower than those observed in mice infected with the isogenic parent strain. Rather, we propose that the significance of these transcriptional effects is dependent on *sarA* repressing the production of extracellular proteases. For example, we previously demonstrated that mutation of *purR* enhances transcription of the genes encoding purine biosynthesis enzymes and *sarA*, the effect of which is to simultaneously enhance the production of these enzymes and limit their protease-mediated degradation (1). This is also not to say that the increased protease production limits the accumulation of the same virulence factors in LAC and UAMS-1 *sarA* mutants, as this seems unlikely given their known differences in cytotoxicity.

Thus, the results we report not only further our efforts to identify virulence factors and regulation pathways of importance in osteomyelitis, but also add to the overall narrative by demonstrating that mutation of the genes encoding Aur and ScpA plays a key role in defining the decreased virulence of LAC and UAMS-1 *sarA* mutants. As assessed by *in vitro* assays, the mutation of *aur/scpA* can reverse the effect of *sarA* mutation to approximately the same extent as complete protease gene mutation, with the mutation of *aur* alone but not *scpA* alone also having a significant impact in some cases. There are several potential explanations for the combined impact of mutating *aur/scpA* despite the lack of significance for *scpA* mutation alone. Cleavage by Aur could expose additional ScpA cleavage sites, or, given the redundancy of *S. aureus* virulence factors, perhaps virulence factor degradation by ScpA can be compensated for unless there is also Aur activity. Most importantly, the results we report support the conclusion that Aur and ScpA play primary roles in defining the reduced virulence of both LAC and UAMS-1 *sarA* mutants in our osteomyelitis model, and that they do so directly by degrading specific virulence factors and thus posttranslationally modifying the virulence factor repertoire. Therefore, this study serves to connect the dots between a history of research into extracellular protease regulation and the modulatory role of extracellular proteases as regulators themselves while expanding the application of extracellular protease-mediated virulence factor degradation to a new strain and model.

## MATERIALS AND METHODS

### Bacterial strains and growth conditions

The mutants used here were generated from LAC and UAMS-1 as previously described (3, 5, 6, 34, 35). Mutants were stored at −80°C in tryptic soy broth (TSB) supplemented with 25% (v/v) glycerol and plated on tryptic soy agar (TSA) containing the appropriate antibiotics for selection at the following concentrations: chloramphenicol, 10 µg/mL; kanamycin, 50 µg/mL; neomycin, 50 µg/mL; erythromycin, 10 µg/mL; spectinomycin, 100 µg/mL; and tetracycline, 5 µg/mL.

### Biofilm formation

Biofilm assays were performed as previously described (3, 6). Briefly, non-tissue culture-treated clear 96-well plates were coated with 20% human plasma in carbonate/bicarbonate buffer for 24 hours overnight at 4°C. After aspiration of the human plasma solution, wells were inoculated with TSB +0.5% glucose +3.0% NaCl (biofilm media, BFM) containing an optical density of 0.05 at 560 nm (OD_560_) of the designated strains or mutants of *Staphylococcus aureus* obtained by dilution of overnight cultures grown in BFM without antibiotic selection. The plates were incubated for 24 hours at 37°C without shaking, then gently aspirated and washed with phosphate buffer solution (PBS) three times. The biofilms were fixed with ethanol and then stained with crystal violet, washed three more times with PBS, and then allowed to dry overnight. Then, crystal violet was eluted with ethanol and the absorbance at 595 nm was read on a FLUOstar Omega plate reader (BMG Labtech) and analyzed by one-way ANOVA with Dunnett’s correction against the *sarA* mutant using GraphPad Prism (version 10.0.0 for Windows, GraphPad Software). A *P*-value ≤ 0.05 was considered statistically significant.

### Conditioned media preparation

Conditioned media (CM) was obtained as previously described (3, 6). Briefly, cultures in TSB were grown overnight and then standardized to an OD_600_ of 8.0. An aliquot was removed, serially diluted, and plated on TSA without antibiotic selection to confirm the cell density of each standardized culture. Bacterial cells were pelleted by centrifugation at 5,000 rpm for 10 minutes, and the supernatant was filter sterilized with 0.20 µm filters.

### Cytotoxicity for mammalian cells

Cytotoxicity of CM was assessed as previously described (3, 6). Briefly, RAW 267.4 (American Type Culture Collection TIB-71) and MC3T3 (ATCC CRL-2594) cells were grown in Dulbecco’s Modified Eagle’s Medium (D-MEM) or Alpha Minimum Essential Medium (αMEM) without ascorbic acid, respectively. Media was supplemented with 10% fetal bovine serum and penicillin and streptomycin (100 µg/mL each). Cells were grown at 37°C in 5% CO_2_ and media was replaced as needed every 2 to 3 days.

For cytotoxicity assays, cells were seeded into black clear-bottom 96-well tissue culture-grade plates at a density of 50,000 cells per well or 10,000 cells per well for RAW 267.4 and MC3T3-E1 cells, respectively. Cells were allowed to form monolayers over 24 hours at 37°C in 5% CO_2_, then the growth medium was replaced with a 1:1 ratio of complete cell culture medium and CM. After another 24 hours, cell viability was assessed using the calcein-AM LIVE/DEAD Viability/Cytotoxicity Kit (Thermo Fisher Scientific) according to the manufacturer’s specifications. Fluorescence intensity was read on FLUOstar Omega plate reader (BMG Labtech) with an excitation wavelength of 485 nm and emission wavelength of 520 nm with the gain set to 95% of the intensity observed in the well that exhibited the highest fluorescence intensity. The cell viability results are reported as the percent (%) viability as determined by the fluorescence intensity of the test sample relative to the intensity of negative control (100% viability), which was a 1:1 ratio of complete media and sterile TSB. A 1:1 mixture of cell culture medium and ethanol (EtOH) was used as a positive control (0% viability). Data were analyzed by one-way ANOVA with Dunnett’s correction against the *sarA* mutant using GraphPad Prism (version 10.0.0 for Windows, GraphPad Software) with a *P*-value ≤ 0.05 considered statistically significant.

### Extracellular protein analysis

The overall profile of proteins present in CM was assessed by SDS-PAGE using 4%–12% gradient Bolt Bis-Tris Plus gels (Thermo Fisher Scientific). Gels were stained with SimplyBlue SafeStain (Thermo-Fischer Scientific) and imaged with a ChemiDoc MP imaging system (Bio-Rad Laboratories). For specific virulence factor analysis, western blots were performed with commercially available rabbit antibodies for staphylococcal protein A (Sigma-Aldrich), leukocidin S (Abcam), leukocidin F (United States Biological), alpha-toxin (Sigma-Aldrich), and Nuc1 (Fisher Scientific) as previously described (2, 8, 36). Uncropped western blot images can be found in supplemental materials (Fig. S1 and S2).

### Murine osteomyelitis model

All experiments involving animals were reviewed and approved by the University of Arkansas for Medical Sciences Institutional Animal Care and Use Committee under animal use protocol number 4124. As previously described (3, 7, 16), mutants for *in vivo* experiments were grown overnight with shaking at 37°C in TSB without antibiotic selection, then washed three times with sterile phosphate-buffered saline (PBS) and resuspended in PBS at a density of 5 × 10^8^ colony-forming units (cfu) per ml, which was confirmed by plating serial dilutions on antibiotic-free TSA. To perform the infections, 6–8-week-old C57BL/6 mice were anesthetized, and the femur of the right hind limb was surgically exposed. A sterile 21-gauge needle was used to drill a unicortical defect in the middle of the exposed femur and 2 µL of bacterial suspension (10^6^ cfu) was then pipetted directly into the medullary canal. Muscle and skin were sutured, and the infection was allowed to proceed for 14 days. Mice were humanely euthanized and tissues harvested. At least two independent experiments with 5 mice per experimental group were done with LAC, UAMS-1, and their isogenic mutants.

### Bacterial burden determination

The femurs were harvested and then frozen so that microcomputed tomography (μCT) could be completed as described below. After imaging, the femurs were homogenized and bacterial burdens were determined as previously described (3) using a Bullet Blender 5 Gold (Next Advance Inc.) and 2 mL of sterile, cold PBS. Serial dilutions of the homogenates were plated on TSA without antibiotic selection for bacterial burden determination. The colony-forming unit (cfu) counts were logarithmically transformed (log(cfu)) so differences in cfu between groups could be assessed using a one-way ANOVA with Dunnett’s correction against the parental strain. Samples with no bacterial counts are reported as having 1 cfu in order to include those samples in the log cfu data set. Analyses were done using GraphPad Prism (version 10.0.0 for Windows, GraphPad Software). Significance was set at *P*-values ≤ 0.05. The bacteria retrieved from *in vivo* experiments were confirmed to be the correct strain by polymerase chain reaction (PCR) analysis to confirm the presence of the *cna* gene in UAMS-1 and its absence in LAC (Forward: CAAGCAGTTATTACACCAGACGG, Reverse: CACCTTTTACAGTACCTTCAATACC), and the presence of the *lukS* gene in LAC and its absence in UAMS-1 (Forward: AATTGCATTGCTTTTGCTATCC, Reverse: ATTTTGAACCATTACCTCCACC).

### Micro-computed tomography (μCT)

Image acquisition was done as previously described (3) using a Skyscan 1275 X-ray Microtomograph (Bruker) with an isotropic voxel size of 6.8 µm and an X-ray voltage of 40 kV (100 μA). Reconstruction was carried out using the Skyscan Nrecon software. The reconstructed cross-sectional slices were processed using the Skyscan CT-analyzer software to perform a semiautomated protocol to create preliminary regions of interest (ROIs) of only cortical bone. The semiautomated protocol was as follows: global thresholding (low = 90; high = 255), round closing in 3D space pixel size 4, round opening in 3D space, pixel size 1, round closing in 3D space, pixel size 8, and round dilation in 3D space pixel size 3. The resulting images were loaded as ROI and corrected by drawing inclusive or exclusive contours on the periosteal surface to keep only the cortical bone. Using these defined ROIs, the volume of cortical bone was calculated using a threshold of 70–255, and the amount of cortical bone destruction was estimated by subtracting the value obtained from each bone from the average obtained from sham-operated bones inoculated with sterile PBS. New bone formation was quantified using the subtractive ROI function on the previously delineated cortical bone ROI images and calculating the bone volume included in the newly defined ROI using a threshold of 45–135. The average value of new bone formation in sham femurs was subtracted from the infected femur values to account for normal new bone formation during the healing process. Statistical analysis was done by one-way ANOVA with Dunnett’s correction for comparison to mice infected with the parent strain. Comparisons were made using GraphPad Prism (version 10.0.0 for Windows, GraphPad Software) with a *P*-value ≤ 0.05 considered statistically significant. For example, µCT images, numbers were randomly assigned to femurs at harvest, and images were generated for the first femurs in each group.

## SUPPLEMENTAL MATERIAL

Fig. S1 and S2 mbio.03383-23-s0001.pdf Uncropped western blots.

ASM does not own the copyrights to Supplemental Material that may be linked to, or accessed through, an article. The authors have granted ASM a non-exclusive, world-wide license to publish the Supplemental Material files. Please contact the corresponding author directly for reuse.
