# Supplementary material for: Increased production of aureolysin and staphopain A is a primary determinant of the reduced virulence of Staphylococcus aureus sarA mutants in osteomyelitis
Source: mBio. 2024 Feb 28;15(4):e03383-23. doi: 10.1128/mbio.03383-23 (PMC11005355; doi:10.1128/mbio.03383-23)
Supplement: Fig. S1 and S2 — Uncropped western blots. [file mbio.03383-23-s0001.pdf]

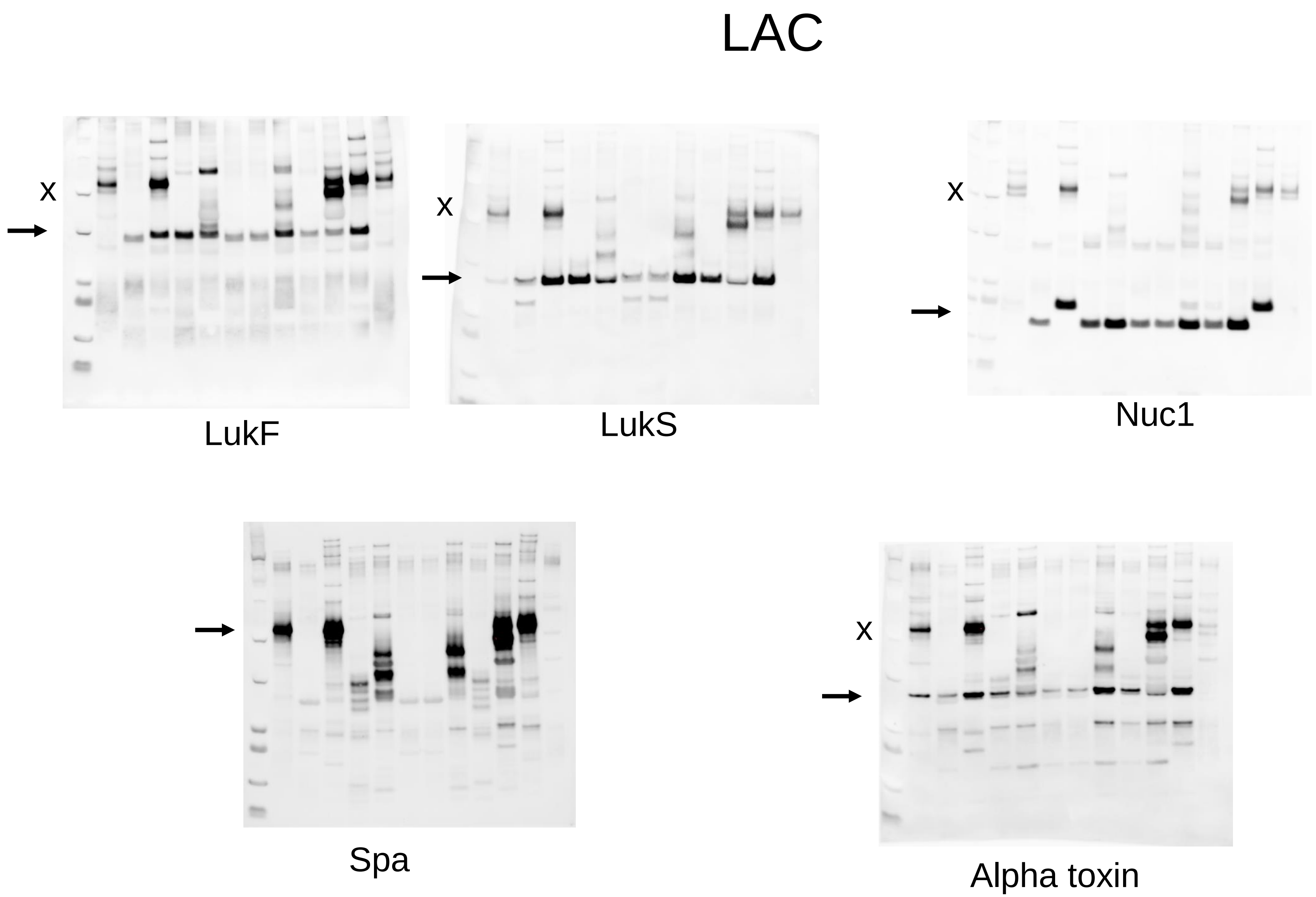

Supplemental Figure 1. Uncropped western images for LAC. Arrows indicate the bands of interest included in the cropped images, “x” indicates bands attributed to Spa in the non-Spa blots, which are visible due to its immunoglobulin binding ability.

UAMS-1

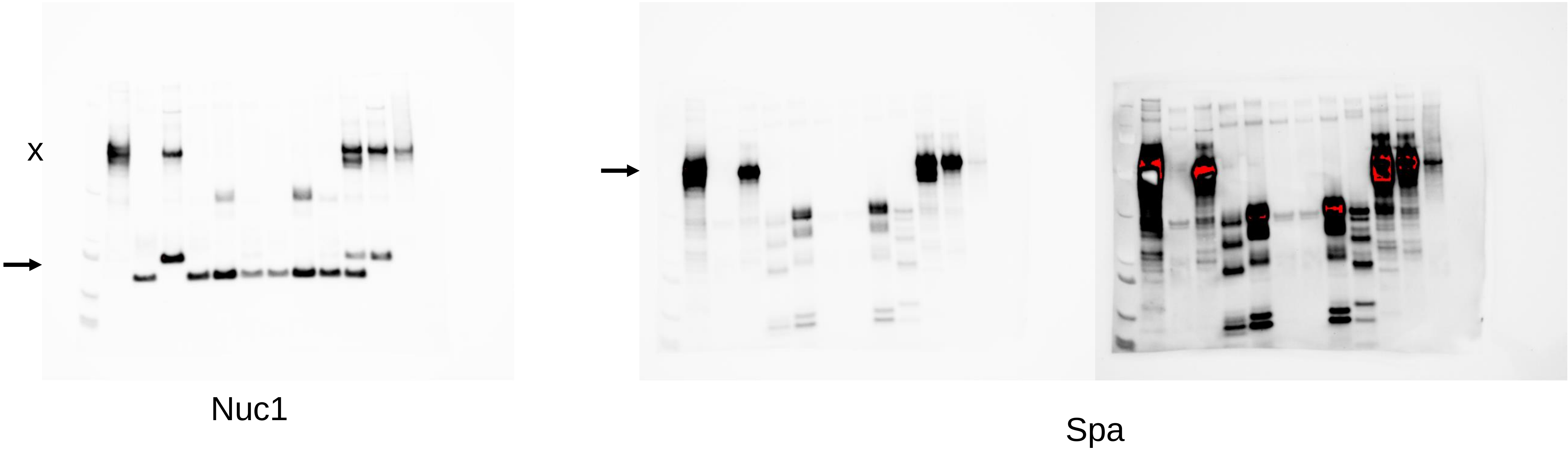

Supplemental Figure 2. Uncropped western images for UAMS-1. Arrows indicate the bands of interest included in the cropped images, “x” indicates bands attributed to Spa in the Nuc1 blot, which is visible due to its immunoglobulin binding ability. The Spa blot is shown with two exposures to ensure the molecular weight markers are visible.
